# HNSCC subverts PBMCs to secrete soluble products that promote tumor cell proliferation

**DOI:** 10.18632/oncotarget.18486

**Published:** 2017-06-15

**Authors:** Marcell Costa de Medeiros, Rajat Banerjee, Min Liu, Giovana Anovazzi, Nisha J. D’Silva, Carlos Rossa Junior

**Affiliations:** ^1^ Department of Diagnosis and Surgery, School of Dentistry at Araraquara, Sao Paulo State University, Araraquara, SP, Brazil; ^2^ Department of Periodontics and Oral Medicine, University of Michigan School of Dentistry, Ann Arbor, Michigan, USA; ^3^ Department of Pathology, University of Michigan Medical School, Ann Arbor, Michigan, USA

**Keywords:** head and neck squamous cell carcinoma, immune response, T cell, immunosuppression and tumor scape, Immunology and Microbiology Section, Immune response, Immunity

## Abstract

The immune system detects shifts from homeostasis and eliminates altered cells. However, neoplastic cells can modulate the host response to escape immunosurveillance thereby allowing tumor progression. Head and neck squamous cell carcinoma (HNSCC) is one of the most immunosuppressive cancers but its role in co-opting the immune system to actively promote tumor growth has not been investigated. In this study, we investigated the influence of soluble factors secreted by HNSCC and non-neoplastic epithelial cells on proliferation, apoptosis, activation, cytokine gene expression and phenotypic polarization of immune cells of healthy donors. Then, we determined if the immunomodulation caused by HNSCC-derived soluble products leads to immunosubversion by assessing proliferation, migration and survival of tumor cells exposed to soluble products secreted by modulated immune cells or co-cultured with immune cells. Soluble products from HNSCC inhibited proliferation and cytokine expression in PBMCs, activation of T cells, and polarization of CD4+ towards the Th17 phenotype. These changes co-opted the immune cells to favor cell proliferation, survival and migration of HNSCC. This immunosubversion was observed both indirectly with secreted products and with direct cell-to-cell contact. We conclude that HNSCC-derived secreted products create an immunosuppressive environment that facilitates evasion of tumor cells and subverts the immune cells into a pro-tumoral phenotype.

## INTRODUCTION

As normal cells undergo malignant transformation, oncogenic phenotypes including proliferation, survival and invasion/migration, are acquired [1]. The immune system detects these shifts from homeostasis and eliminates altered cells; however neoplastic cells modulate the host response to escape this immunosurveillance thereby promoting tumor development and progression [1, 2]. In fact, evasion of immunosurveillance is one of the eight hallmarks of cancer, which also include proliferation, survival, invasion and metastasis, angiogenesis, reprogramming energy metabolism, evasion of growth suppression, and stemness [1]. Importantly, tumors ‘proactively’ influence the immune response to foster growth. This modulation of the immune system by tumors may either result in the inactivation of the immune response (‘immunoescape’) [3] or in the activation of the immune system [4] in a subversion of the defense response to increase blood supply to the growing tumor mass and/or to facilitate its local invasion or metastatic dissemination. Some cancers grow despite the immune response (inflammation) in the microenvironment (immune evasion); whereas other cancers grow faster in the presence of an immune response (immunosubversion). There are also cancers that inhibit the immune response (immunosupression) to facilitate tumor growth and invasion. All these instances illustrate the complexity of tumor-immune system interactions, which in solid tumors are mainly dictated by the tumor microenvironment.

Head and neck squamous cell carcinoma (HNSCC), the 6^th^ most common cancer in the world, is one of the most immunosuppressive cancers [5-8]. It is a lethal disease and is the 5^th^ most common cause of cancer-related deaths [9]. The significance of immunosurveillance in HNSCC is highlighted in mouse and human studies; in mouse models of chemically-induced oral cancer, T-cell deficient animals presented a decreased latency for tumor development and an increase in the number of lesions [10], indicating the protective role of adaptive immunity. In high-throughput gene array analysis of HNSCC, increased expression of genes that dampen the immune response, such as IL-4, has been detected [11]. This tumor-mediated modulation of immunity is demonstrated by an overall decrease of CD4+ T-cells coupled with a shift to a T helper-2 phenotype (increased expression of IL-4 and reduced expression of IL-2) in HNSCC patients [12]. Increased levels of biological mediators such as IL-4, IL-10 and TGF-b, present both in HNSCC [13, 14] and in the microenvironment of other tumors [15-17] may affect the adaptive immune response, thereby suppressing immune-surveillance and facilitating tumor progression [18].

There are two possible mechanisms associated with tumor evasion of adaptive immunity: 1) depletion of T cells thereby inhibiting detection of tumor cells; and 2) induction of immune tolerance. In support of the first mechanism, the increased production of Fas ligand by tumor cells [18] may induce apoptosis of immune cells; T cells exposed to metastatic HNSCC were more prone to apoptosis [19]. However, the ineffectiveness of cytokine-induced stimulation of immunity and of transfer of effector T-cells as therapeutic approaches in HNSCC [20, 21], coupled with evidence that indicates that T-cell in the tumor microenvironment become suppressed or inactivated [22], point to the likelihood of a second putative mechanism.

An increase in tumor-infiltrating T cells is positively correlated with better prognosis and response to treatment in multiple cancers, including ovarian and colon cancer [23, 24]. In early-stage tongue cancer, CD8+ T cells and NK cells infiltrating the tumor nest present a predominantly suppressed phenotype, whereas the cells in the surrounding tissue stroma display more active phenotypes [25]. Increased presence of activated (CD4+CD69+) and regulatory (CD4+FoxP3+) T helper cells were both associated with better outcomes of HNSCC, better prognosis and better locoregional control of the disease, respectively [26]. This apparently contradictory finding of a ‘double-edged immunological sword’ is conceptually interpreted based on the central role of CD4+ cells in the anti-tumor response [27, 28]. On one hand, active CD4+ T cells are needed for proper activity, memory and sustained function of cytotoxic T cells [29, 30]. Activated CD4+ cells can also exert cytotoxic-independent anti-tumor activities, by recruiting other effector cells such as macrophages and eosinophils [31] or by direct lysis of MHC class II-positive T cells [32]. On the other hand, besides the protective effect of an activated immune response, increased prevalence of infiltrating CD4+ cells with a regulatory (immunosuppressive) phenotype (CD4+CD25+FoxP3+) is also associated with better prognosis in HNSCC. The rationale in this case is that Treg cells dampen the immune response, reducing inflammation and, consequently, preventing extracellular matrix degradation and tumor growth and invasion [33, 34]. It is important to note that activated T helper cells (CD4+CD69+) may include cells with Th1 (‘inflammatory’ / anti-tumor) or Th2-type (less inflammatory/humoral-type response) phenotypes, and the relevance of enhanced or suppressive immunity may vary with type of cancer and with the stage of tumor development [26]. Collectively, this evidence indicates that the relative numbers of specific phenotypes of tumor-infiltrating and circulating T-cells is considered of prognostic value in HNSCC [26, 35, 36].

Although HNSCC is well established as an immunosuppressive cancer, immunosubversion, or the impact of secreted factors from the modulated immune system on tumor proliferation, survival and migration, has not been extensively investigated. The data we present in this study indicates that HNSCC can harness the immune system to promote tumor progression. Besides tumor-secreted cytokines, the factors secreted by the immune system also have an important role HNSCC progression [37]. For example, immune cells may secrete pro-angiogenic cytokines to promote tumor angiogenesis, a hallmark of cancer [1, 37]. However, the possibility of a direct role of secreted factors from the immune system in promoting HNSCC progression has not been investigated. The goals of this study were to assess the impact of secreted products of HNSCC cells on normal immune cells, and to evaluate if this modulation alters secreted products from the immune cells to promote tumor progression.

## RESULTS

### HNSCC-derived soluble products inhibit proliferation of PBMCs

**Figure 1:**
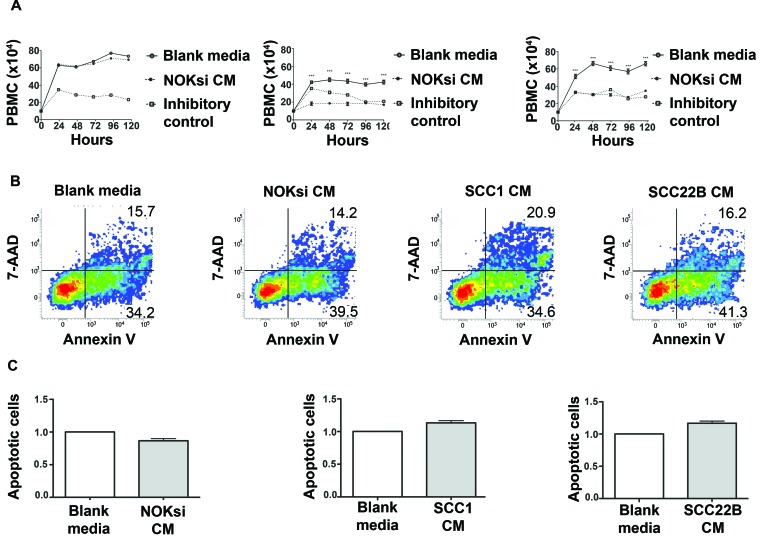
Products secreted from head and neck cancer cells inhibit proliferation of PBMCs **A.** PBMC proliferation over 120h was determined by direct counting of cells using the trypan blue dye exclusion assay. PBMCs were cultured in the presence and absence of CM from NOKsi, UM-SCC-1 and UM-SCC-22B cell lines (left to right). Negative controls were performed in parallel using blank RPMI1640 medium maintained in the incubator for the same length of time as the CM. Mitomycin C, 10 ug/ml, inhibits proliferation of PBMCs and was used as an inhibitory control for the assay. **B.** Representative dot-plots of Annexin V/7-AAD apoptosis assays performed at the 48h period. **C.** Apoptosis of PBMCs treated with blank or conditioned medium. Mean and standard deviation of the fold change in the proportion of total apoptotic PBMC cells (early + late-stage apoptosis) treated with CM from NOKsi, UM-SCC-1 and UM-SCC-22B (left to right). ***p < 0,001.

To determine the impact of HNSCC on proliferation of PBMCs, these cells were stimulated with conditioned medium (CM) from UM-SCC-1 and UM-SCC-22B, or a non-neoplastic cell line (NOKsi). In contrast to CM from NOKsi, CM from UM-SCC-1 and UM-SCC-22B significantly inhibited proliferation of PBMCs (Figure 1A). To verify that the inhibitory effect was not due to apoptosis of PBMCs, apoptosis was quantified. Notably, the SCC-mediated inhibitory effect on proliferation of PBMCs was not accompanied by a significant increase in apoptosis, assessed at both 48h (Figure 1B, 1C) and 120 h (data not shown).

### HNSCC reduces activation of CD3 and CD8 cells

**Figure 2:**
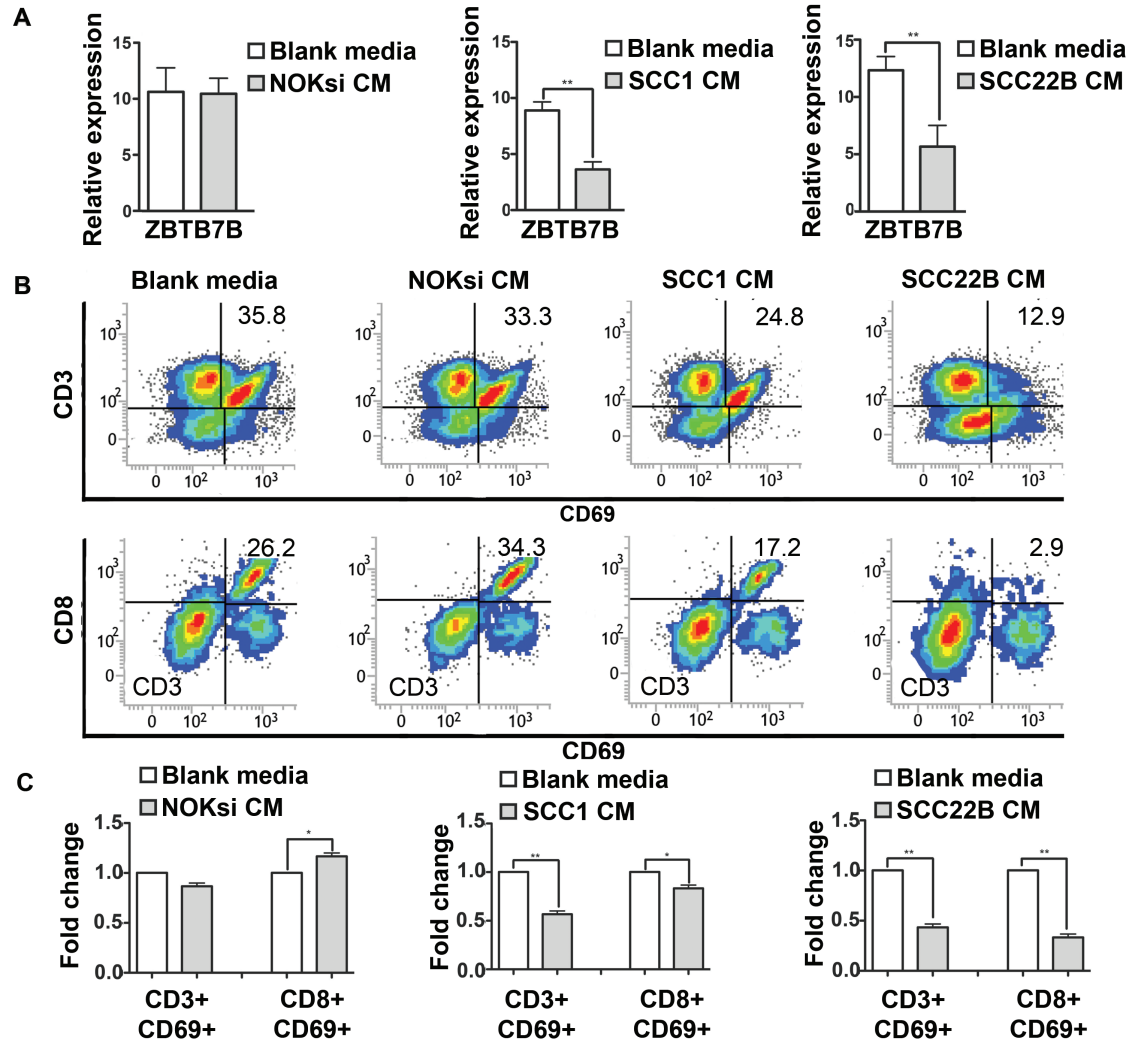
Secreted products from HNSCC decrease activation of CD3 and CD8 cells PBMCs were stimulated with CM from NOKsi, UM-SCC-1 and UM-SCC-22B (or Blank media RPMI1640) for 96h. **A.** RT-qPCR for expression of ZBTB7B gene (immature T cells) in PBMCs treated with CM of NOKsi, UM-SCC-1 and UM-SCC-22B. **B.** Representative dot-plots of the proportion of CD3+ and CD8+ cells expressing CD69 (marker of activation). **C.** Fold change of the proportion of CD3+ and CD8+ cells expressing CD69 activation. *p < 0,05, **p < 0,01.

To determine the impact on T cells, PBMCs were cultured in the presence of CM from HNSCC cells. The Zinc Finger and BTB Domain Containing 7B (ZBTB7B) gene encodes a transcription factor that is a key regulator of commitment of immature T cells. Its expression is both necessary and sufficient for CD4 lineage commitment whereas its absence drives commitment to CD8 cells [38]. PBMCs exhibited reduced expression of ZBTB7B after exposure to CM from HNSCC (Figure 2A). CM from HNSCC also significantly reduced the expression of the activation marker CD69 in both CD3+ and CD8+ cells (Figure 2B, 2C).

### HNSCC-derived soluble products suppress Th17 phenotype

**Figure 3:**
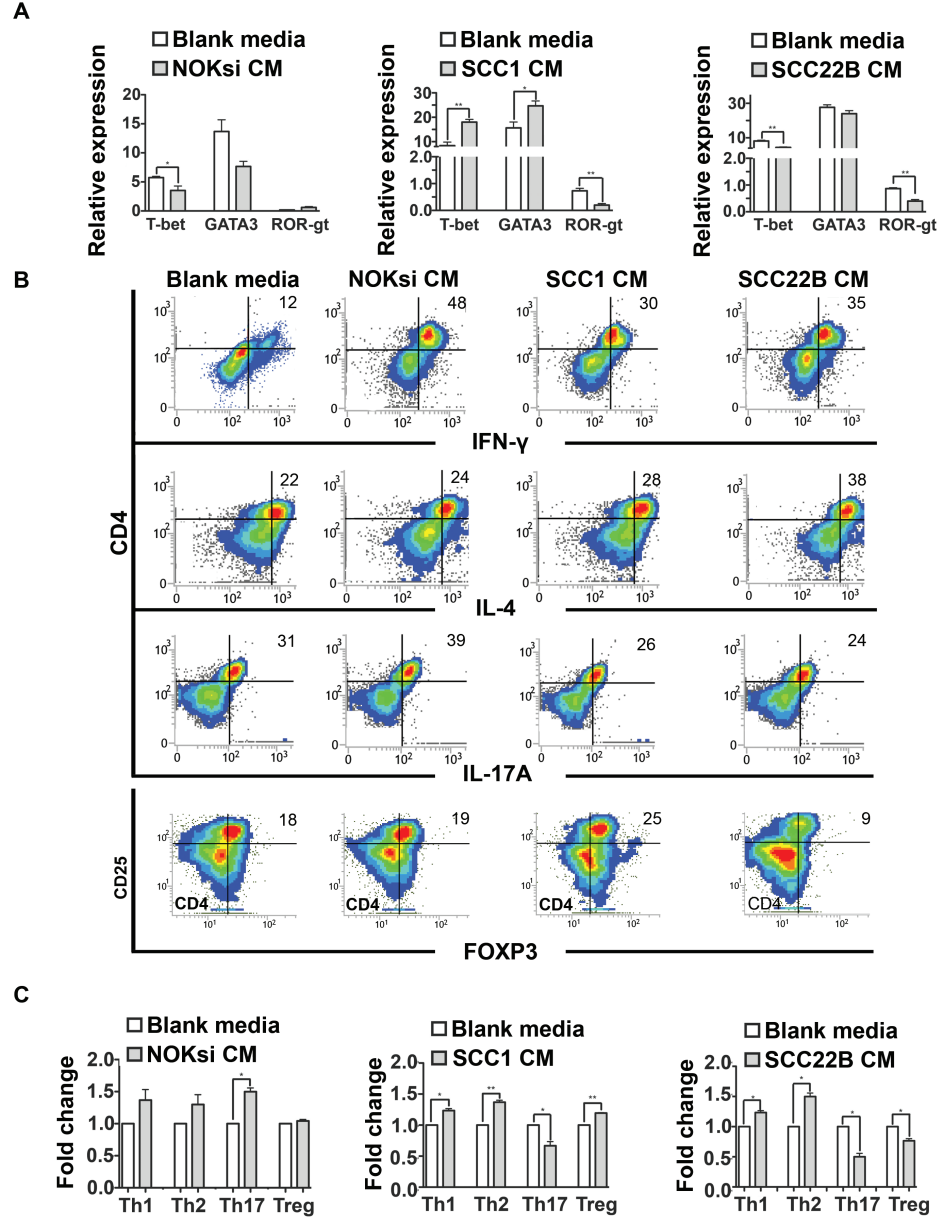
HNSCC-derived cytokines inhibit Th17 **A.** Gene expression of transcription factors associated with Th1, Th2 and Th17 phenotypes (T-bet, GATA3 and ROR-gt respectively) of PBMC stimulated for 96h with CM of NOKsi, UM-SCC-1 and UM-SCC-22B assessed by RT-qPCR (left to right). **B.** Representative dot-plots of the immunophenotyping of CD4+ cells after stimulation for 96h with blank media and CM from NOKsi, UM-SCC-1 and UM-SCC-22B (left to right) assessed by flow cytometry. **C.** Fold change of the proportion of the CD4 phenotypes in each experimental condition in comparison to control (RPMI1640). *p < 0,05, **p < 0,01, ***p < 0,001.

**Figure 4:**
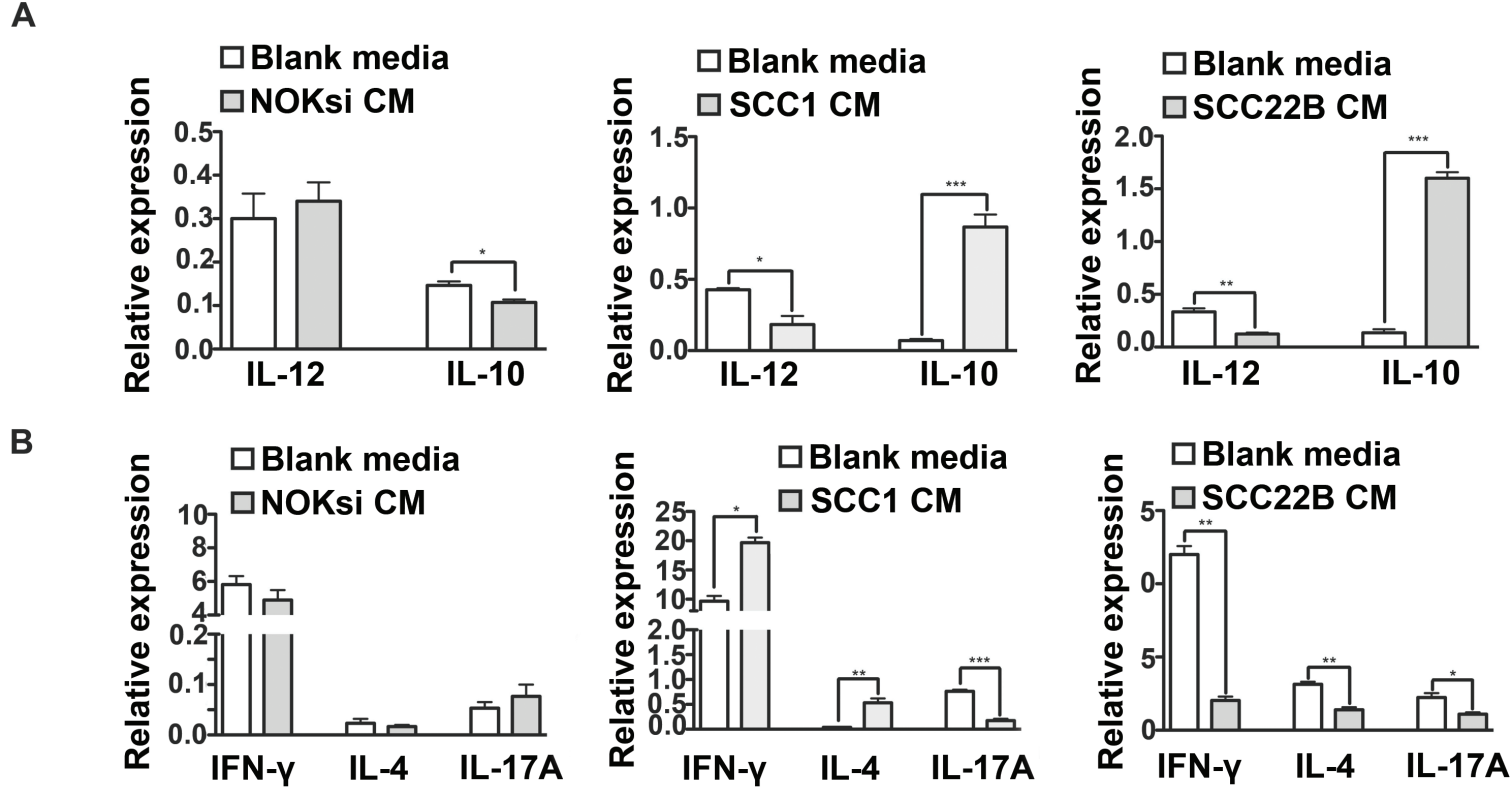
HNSCC soluble products inhibit gene expression of pro-inflammatory cytokines by PBMCs PBMCs were cultured in the presence of CM from NOKsi, UM-SCC-1 and UM-SCC-22B or with Blank media for 96h. Total RNA was isolated and gene expression of selected cytokines genes was assessed by RT-qPCR. Data is presented as mean and standard deviations of normalized gene expression, including: **A.** IL-12 and IL-10, correlated with the monocyte response and **B.** IFN-g, IL-4 and IL-17A, correlated with Th1, Th2 and Th17 phenotypes, respectively. *p < 0,05, **p < 0,01.

Th17 is the most anti-tumoral phenotype of T-cells [39, 40]. Exposure of CD4+ T-cells to CM from HNSCC for 96h resulted in a significant decrease of gene expression of nuclear receptor ROR- t (ROR-gt), which was supported by the significant decrease of the percentage of Th17 cells (CD4+/IL17A+) (Figure 3A-3C). In contrast, there was an increase in polarization towards the Th17 phenotype when PBMCs were cultured in the presence of CM from the control non-neoplastic cell line NOKsi. Polarization towards Th1 and Th2 phenotypes assessed by flow cytometry was significantly increased when PBMCs were cultured in the presence of CM from both HNSCC cell lines; however the magnitude of the increase of Th2 phenotype was greater than that of Th1. The percentage of polarization towards the Treg phenotype was differentially modulated between HNSCC cell lines: increased in the presence of CM from UM-SCC-1 and decreased in the presence of UM-SCC-22B (Figure 3B, 3C). Other representative Th1/Th2-cytokines were analyzed by RT-qPCR. Expression of IL-12 was markedly decreased, whilst IL-10 expression increased after exposure to CM from both HNSCC cell lines (Figure 4A). Expression of some cytokines (IFN-g and IL-4) was not consistent with Th-type response, however there was a consistent reduction in IL-17A expression by RT-qPCR in PBMCs stimulated with CM from both HNSCC cell lines (Figure 4B). These findings indicate an immunosuppressive effect caused by exposure of PBMCs to CM from HNSCC cells, characterized by the downregulation of pro-inflammatory and upregulation of anti-inflammatory cytokines/phenotypes.

### Soluble products from HNSCC-modulated PBMCs promote proliferation of HNSCC

**Figure 5:**
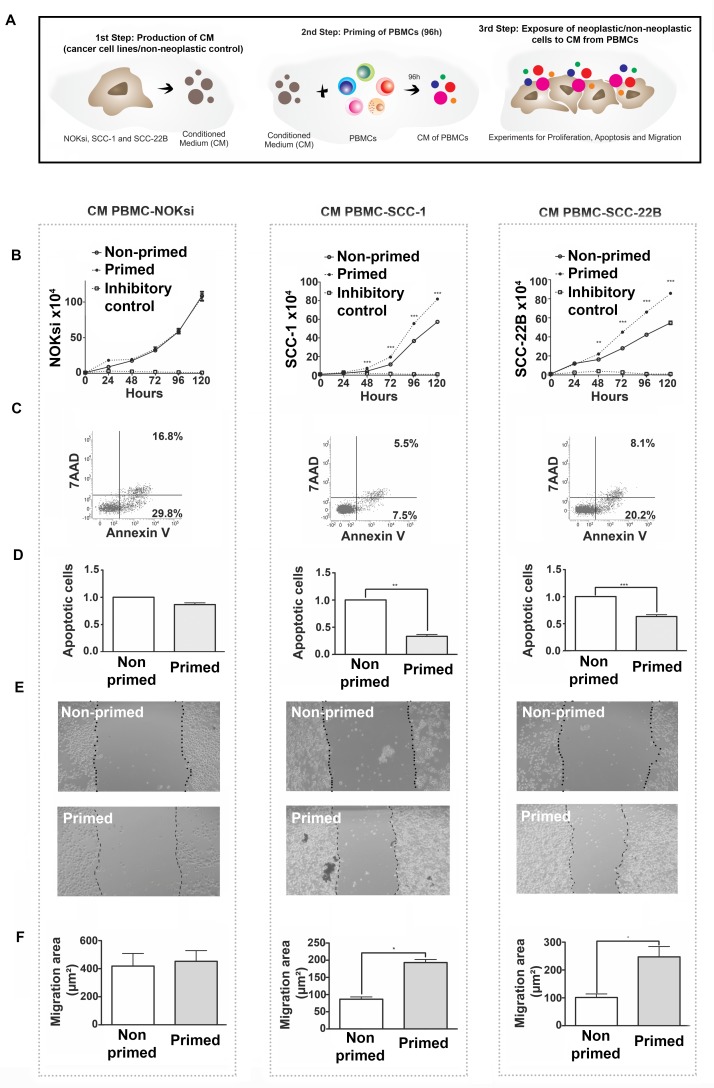
Immunomodulation of PBMCs by HNSCCs (‘priming of PBMCs’) leads to pro-oncogenic effects independent of direct contact **A.** Schematic of experiments. **B.** Proliferation of NOKsi, UM-SCC-1 and UM-SCC-22B cell lines cultured in the presence of conditioned media (CM) from non-primed (CM PBMC-RPMI, negative control) or primed PBMCs (i.e. from PBMCs incubated for 96h with CM from NOKsi, UM-SCC-1 or UM-SCC-22B cells). **C.** Representative dot-plots of the apoptosis assays of NOKsi, UM-SCC-1 and UM-SCC-22B cells after 48h of exposure to CM from non-primed and primed PBMCs. **D.** Mean and standard deviation of the fold change of apoptotic NOKsi, UM-SCC-1 and UM-SCC-22B cells after 48h exposure to CM from non-primed (control) and primed PBMCs. **E.** Representative images (40X magnification) of the migration assay (24h) of NOKsi, UM-SCC-1 and UM-SCC-22B cells exposed to CM from non-primed (control - CM PBMC-RPMI) and primed PMBCs. **F.** Quantification of the migrated area for each cell line (NOKsi, UM-SCC-1 and UM-SCC-22B). *p < 0,05, **p < 0,01, ***p < 0,001.

PBMCs were incubated (‘primed’) with CM from HNSCC or NOKsi cells and then CM was generated from these primed PBMCs. PBMCs were initially exposed to CM from non-neoplastic and HNSCC cells for 96h. The supernatant (CM) of these primed/exposed PBMCs was collected and used to treat the respective non-neoplastic and HNSCC cell lines (schematic in Figure 5A). Cell proliferation was studied in the presence and absence of the CM from primed PBMCs. There was no effect on proliferation of NOKsi cells; however exposure to PBMC CM significantly increased the proliferation of both HNSCC cell lines (UM-SCC-1 and UM-SCC-22B) (Figure 5B). The increase in proliferation occurred concurrently with a significant reduction in apoptosis of HNSCC cells (Figure 5C and 5D, middle and right panels). CM from PBMCs did not affect apoptosis of NOKsi cells (Figure 5C and 5D, left panels). CM from primed PBMCs also increased migration of both HNSCC cell lines (Figure 5E and 5F, middle and right panels). These findings demonstrate an effective immunosubversion of immune cells, since priming PBMCs with secreted products from HNSCC cells modulates the immune cells to secrete products that enhance proliferation, survival, and migration of tumor cells.

### HNSCC-modulated PBMCs enhance proliferation of tumor cells in co-culture

**Figure 6:**
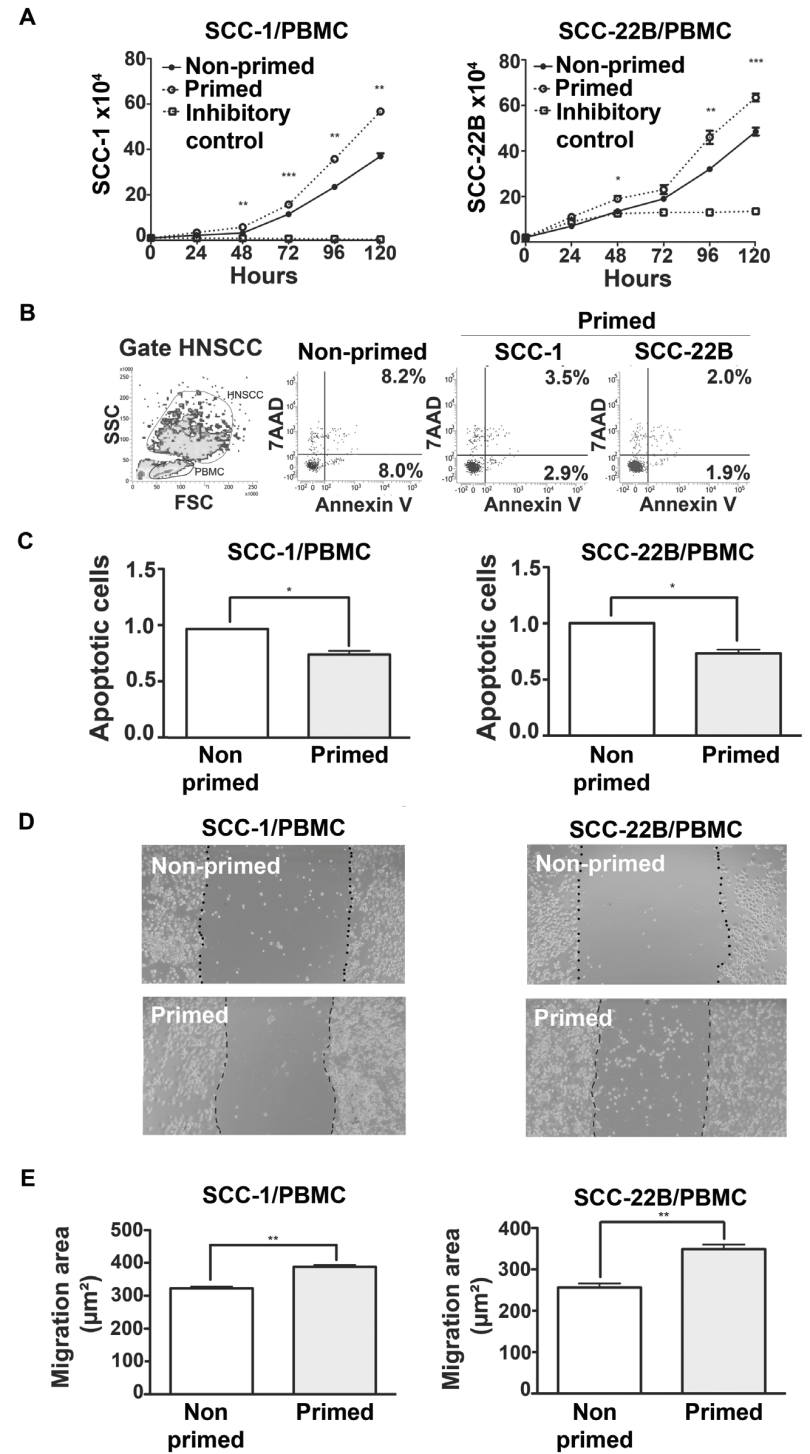
Direct contact between primed PBMCs and HNSCC has pro-tumor effects **A.** HNSCC proliferation after co-culture with non-primed (RPMI) and primed (CM) PBMC in the proportion 1:5 (PBMC:HNSCC). **B.** Dot-plot of FSCxSCC indicating the gating strategy to distinguish HNSCC from PBMC cells. Representative dot-plots of apoptosis of HNSCC cells, 48h after initiation of co-cultures with PBMCs. Non-primed dot-plot is representative of SCC-1 and SCC-22B co-culture with PBMC incubated with RPMI only. **C.** Mean and standard deviation of the fold change on the percentage of apoptotic HNSCC cells after 48h of co-culture with non-primed (Control, RPMI) and primed PBMCs. **D.** Representative images (40X magnification) of the migration of HNSCC cell lines after 24h in co-culture with non-primed (Control, RPMI) and primed PBMCs. **E.** Quantification of the migrated area of each HNSCC cell line in co-culture with non-primed (control, RMPI) and primed PBMCs. *p < 0,05, **p < 0,01, ***p < 0,001.

To assess if subversion of the immune cells caused by HNSCC secreted products also affects direct cell-to-cell contact, PBMCs primed with CM from two HNSCC cell lines (UM-SCC-1, UM-SCC-22B) for 48h were co-cultured with the respective HNSCC cell line. Proliferation of HNSCC co-cultured with primed PBMCs was significantly greater than HNSCCs co-cultured with non-primed PBMCs (cultured in regular medium for 48h prior to the co-culture) (Figure 6A). Moreover, apoptosis in both HNSCC cell lines was reduced when co-cultured with primed PBMCs compared to co-culture with non-primed PBMCs (Figure 6B and 6C). Migration of both HNSCC cell lines was also significantly enhanced when co-cultured with primed PBMCs (Figure 6D and 6E). Collectively these data show that HNSCC-modulated PBMCs confer an advantage to proliferation, survival and migration of HNSCC.

## DISCUSSION

The goals of this study were to assess the overall impact of secreted products of HNSCC on immune cells, and to evaluate if this modulation alters secreted products from the immune cells to promote tumor progression. Our results demonstrate that soluble products secreted by HNSCC have immunosuppressive properties; in another example of immunosubversion, the modulated immune cells enhance proliferation, survival and migration of HNSCC, both via immune cell-secreted products and by cell-to-cell contact.

Proliferation of PBMCs is significantly inhibited by soluble products derived from HNSCC cell lines, but not by soluble products from a non-neoplastic oral keratinocyte cell line. Interestingly, the HNSCC-induced decrease in proliferation of PBMCs was not due to apoptosis of PBMCs, which suggests an effect on the cell cycle. Exosomes from breast cancer cell lines reduced proliferation of activated murine splenocytes, an inhibition attributed to TGFβ present within exosomes [41]. Proliferation of activated lymphocytes from healthy donors was also decreased when exposed to secreted products from a cervical cancer cell line [42]; however, in contrast to our results, this reduction in the proliferation of the lymphocytes was associated with increased apoptotic cell death.

Besides the decrease in proliferation, soluble products of HNSCC cell lines reduced the expression of the ZBTB7B gene. This may inhibit the commitment of näive T cells towards the T helper (CD4+) phenotype and is associated with an enhanced commitment of näive T cells towards a CD8+ phenotype, which would have an anti-tumor effect [43]. In fact, the number of CD8+ cells infiltrating the tumor, in the surrounding stroma or even in the peripheral circulation is positively associated with a positive prognosis / improved outcome in pre-clinical and clinical studies [44-49]. However, consistent with an immunosuppressive effect exerted by secreted products from HNSCC, we observed a decrease on the activation status of CD8+ cells.

The phenotype of CD4+ cells was also affected by soluble products derived from HNSCC cells. In contrast with other studies [47, 50-52], we observed an increase on Th1 phenotype in response to the secreted products from HNSCC cells. The percentage of CD4+ cells polarized to the Th2 phenotype was also significantly increased and, in fact, the relative magnitude of the increase in the proportion of Th2 cells was greater than the increase in the proportion of Th1-polarized cells. This suggests a net effect towards a predominantly Th2-type phenotype. A relative skewing towards Th2 has been reported in HNSCC and other cancers [7, 51, 53-55]. In HNSCC, the skew towards a Th2 phenotype is associated with worse prognosis and response to treatment [56-58].

Interestingly, exposure to secreted products from both HNSCC cell lines used in this study consistently and significantly inhibited the proportion of cells polarized to the Th17 phenotype. In pre-clinical models, progression from premalignant to malignant lesions is associated with a decrease in the Th17-type response and is accompanied by an increase on Treg cells [59, 60]. However, there is contradictory information regarding the Th17 phenotype in HNSCC. Patients with node involvement in metastatic HNSCC present a reduced prevalence of Th17 cells [61]; whereas other studies show an increase in the prevalence of Th17 cells in the peripheral circulation of HNSCC patients [62, 63]. In the present study, inhibition of Th17 polarization is consistent with the immunosuppressive effects of secreted products of HNSCC.

The proportion of immunosuppressive Treg cells was differentially affected by secreted products from different HNSCC cell lines used in this study: products secreted by UM-SCC-1 increased the prevalence of Tregs, whereas products secreted by UM-SCC-22B decreased the prevalence of Tregs. This finding may reflect the heterogeneity of HNSCC, particularly the variations in the tumor microenvironment and the consequences on the modulation of the immune response. Studies correlate a high prevalence of Tregs (FOXP3+) with advanced or synchronous tumors [64-66]. Patients with HNSCC present a greater number of circulating Tregs in comparison with healthy cohorts [67]; however the frequency of Tregs in patients with oral cavity squamous cell carcinoma and healthy cohorts was similar [67]. The prevalence of Tregs is also correlated with the stage of HNSCC, as a greater number of Treg suppressor cells in the peripheral blood of patients with late stage laryngeal tumors, in comparison with patients with earlier stages and patients with oropharyngeal tumors [68].

The heterogeneity of HNSCC may be related to the prevalence of distinct Treg subtypes (resting Tregs, activated Tregs, and cytokine-secreting CD45RA-Foxp3^low^CD4+ T cells) in the peripheral circulation. Active, cytokine-secreting Tregs were increased and resting Tregs were reduced in patients with laryngeal squamous cell carcinoma [47]. Moreover, the prevalence of activated, cytokine-secreting Tregs was positively correlated with tumor stage and nodal status [47].

Secreted products from HNSCC inhibited gene expression of pro-inflammatory cytokines by PBMCs, notably expression of IL-17A and IL-12, whereas IL-10 was increased. Expression of both interferon-g and IL-4 were increased after exposure to soluble products from UM-SCC-1, which is consistent with the increase in Th1 and Th2 phenotypes. However, soluble products from UM-SCC-22B markedly decreased the gene expression of both interferon-g and IL-4, which is inconsistent with the increase in Th1 and Th2 phenotypes observed. These findings may be limited by the lack of mRNA-protein correlation, but may also be related to the regulation of these cytokine genes in immune cell types other than T cells, which are present in the PBMC population. Overall, the downregulation of gene expression of inflammatory cytokines, in particular of IL-12, accompanied by the increased expression of anti-inflammatory IL-10 indicates the immunosuppressive effects of HNSCC soluble products and is suggestive of a polarization of monocytic/macrophagic response towards the alternative activation or ‘M2’ phenotype. IL-12 serum levels were reduced and IL-10 serum levels were elevated in patients with HNSCC in comparison with a healthy control cohort [69]. The regulation of IL-10 expression by soluble products of HNSCC may be especially relevant, as the serum levels of IL-10 are increased in patients with dysplastic lesions and even greater in patients with HNSCC [53]. Moreover, IL-10 levels were greater in patients with hypopharyngeal and laryngeal tumors, particularly in advanced stages (T3 and T4) [69].

The immunosuppression associated with some types of cancer is thought to favor tumor growth and invasion by allowing the evasion of tumor cells from immunosurveillance. Priming immune cells with soluble products from HNSCC cell lines resulted in modulation of the secreted products from these immune cells, which caused an increase in proliferation, survival and migration of HNSCC cells. Strikingly, even in co-culture with direct cell-to-cell contact, the primed immune cells increased tumor cell proliferation, survival and invasion. Thus, HNSCC subverts immune cells to favor tumor growth (‘immunosubversion’) [70]. The findings in this study are especially indicative of immunosubversion by soluble products of HNSCC, since the HNSCC and immune cells were derived from different individuals, which would allow for detection of cancer cells by variations in class I MHC molecules. Even though cancer cells may lose the expression of class I MHC thereby escaping detection by CD8+ cells, NK cells would be able to detect and eliminate the cancer cells [71, 72]. Non-primed (or ‘non-subverted’) PBMCs reduce proliferation and induce apoptotic cell death of co-cultured lung cancer cells [73] and interfere with downregulation of class I MHC in melanoma, kidney and lung cancer cell lines [74]. In contrast, immune cells themselves may trigger immunosuppression associated with cancer, as shown recently in co-culture of immune and liver cancer cells, in which immune cells induce tumor cells to produce Indoleamine 2, 3-Dioxygenase 1 (IDO1) [75], which induces T cell anergy and promotes polarization of CD4+ cells to the immunosuppressive Treg phenotype.

The experimental approach to assess the immunomodulatory properties of cancer cell-secreted products has not been previously used in the context of HNSCC. The intent of this study was to assess the overall impact of tumor cell-secreted products on normal immune cells, since the main goal was to assess how the tumor microenvironment affects immune cells, and not to identify which factor is more relevant in this modulation. In the highly complex tumor microenvironment and considering the overlapping and even redundant role of various cytokines, inhibition or blocking a single cytokine is likely to have little effect on the modulation of immune cells. Knowledge derived from this study may be useful for immuno-targeted therapies, focused on restoring Th17 cells, for example. We used PBMCs from the same two donors for all experiments for consistency purposes; however this limits the range of immune cell responses that are influenced by genetic variations in different individuals. Similarly, we used two HNSCC cell lines and a non-neoplastic epithelial cell line derived from human oral mucosa as control, which needs to be considered in view of the heterogeneity that is peculiar to HNSCC. Finally, other cell types in the tumor microenvironment, particularly cancer-associated fibroblasts (CAFs) and endothelial cells, were not assessed in this study. Also, in the experimental design used in this study we cannot rule out the possible role of extracellular vesicles (EV) in the immunomodulatory effects.

In summary, our study shows that HNSCC induces prominent immunosuppression and co-opts immune cells to promote tumor cell proliferation, survival and migration.

## MATERIALS AND METHODS

### Cell culture

Commercially obtained PBMCs (CTL, Cellular technologies, OH, USA) from two healthy donors were used in all experiments. Cells were thawed according to the supplier’s instructions immediately prior to use. PBMCs were grown in RPMI1640 (Gibco, Cat# 11875119) supplemented with penicillin (100 UI/mL), streptomycin (100 ug/mL) (Gibco, Cat# 15140163) and heat-inactivated FBS (10%) (Gibco, Cat# 160000). Viability of the PBMCs was checked immediately before each experiment and was always higher than 90%. For all experiments PBMCs were stimulated with rhIL-2 (10 ng/mL, R&D Systems, Cat# P60568), concanavalin A (2.5 ug/mL, Cat# C5275) and phytohemagglutinin (2.5 ug/mL, Cat# L1668, both Sigma-Aldrich,). PBMCs were stimulated with CM from HNSCC and NOKsi cell lines in a proportion of 2:1 (CM:RPMI) in all experiments.

HNSCC cell lines UM-SCC-1 (oral cavity, Male, T2N0M0) [76] and UM-SSC-22B (metastatic lymph node of tumor primary localized in hypopharynx, Female, T2N1M0) [76, 77] were provided by Dr. Thomas Carey (University of Michigan) and the non-neoplastic cell line, NOKsi [78], was provided by Dr. Silvio Gutkind (NIDCR/NIH). Cells were cultured in DMEM (Gibco, Cat# 11965)/10% FBS supplemented with penicillin and streptomycin.

### Conditioned medium

(CM) from unstimulated NOKsi, UM-SCC-1 and UM-SCC-22B were used as the source of constitutively-produced soluble secreted products to stimulate PBMCs. Briefly, 2x10^6^ cells were grown in complete medium for 24h to reach a confluence of 60-70% and washed with non-supplemented RPMI media (Gibco Cat# 11875119) for 8h. Subsequently, cells were incubated in 4mL of RPMI for 16h. Supernatant was collected and centrifuged at 10,000 RPM for 10 min. This CM was aliquoted and stored at -80^o^C for less than 30 days. Aliquots were thawed on ice only once, immediately prior to use.

### Experiments with primed PBMCs

Priming of PBMCs to generate both PBMC-CM (for indirect experiments/ effect of soluble immune cell products) and primed PBMCs (for direct co-culture/ effect of cell-to-cell contact) was performed by incubating freshly-thawed PBMCs with CM from HNSCC or NOKsi for 96h. Briefly, 1x10^6^ PBMCs were plated in each well of a 12-well plate using a mixture of RPMI1640 and CM (1:2). After 96h the supernatant was processed and stored as above. PBMCs for direct co-culture experiments were harvested, washed once in PBS, counted on a hemocytometer and used immediately. PBMCs were incubated with blank RPMI1640 culture medium using the same protocol to generate the blank control and non-primed PBMCs. CM from non-primed PBMCs and also non-primed PBMCs were prepared simultaneously and used as negative controls. (Figure 5A).

We designated the CM from PBMCs as ‘CM-PBMC-X’, where ‘X’ refers to the CM used to prime the PBMCs. For example, ‘CM-PBMC-RPMI’ refers to CM from non-primed PBMCs incubated with blank medium; ‘CM-PBMC-NOKsi’ refers to CM from PBMCs primed with CM from NOKsi cells. For direct co-culture experiments (Figure 6), HNSCC cells were cultured with primed PBMCs in the proportion of 1:5, PBMCs:HNSCC.

To assess indirect (PBMC-CM) and direct (co-culture) effects of primed PBMCs, HNSCC and NOKsi cells were plated in 24-well plates at a density of 1x10^4^ cells for proliferation and apoptosis assays. For migration assays, 1x10^5^ cells were plated in 24-well plates the day before the experiment. The same outcomes were assessed in both indirect (PBMC-CM) and direct (co-culture) experiments: proliferation, apoptosis and migration of HNSCC and NOKsi cell lines.

### Proliferation and apoptosis

Proliferation was assessed in a hemocytometer with 0.4% trypan blue at 24, 48, 72, 96 and 120h. Three independent experiments were performed with four replicates in each experiment for each time point/condition. In co-culture experiments, PBMCs and HNSCC were distinguished visually by size.

Apoptosis was determined by the Annexin V/7-AAD assay (PE Annexin V Apoptosis Detection Kit I, cat#559763, BD Biosciences) according to the supplier’s instructions using a BD FACSVerse cytometer (BD Bioscience). Briefly, supernatant with non-attached cells, and adherent cells dissociated for 10 min with enzyme-free dissociation buffer (Gibco cat#13151014), were combined, centrifuged for 5 min at 400x*g* and apoptosis was quantified. Apoptosis was assessed at 48h when changes in proliferation were already significant, and at 120h (data not shown). In co-culture experiments, PBMCs were distinguished from HNSCC cells by size, creating a gate on the FSC x SSC dot-plot.

### Cell migration

*In vitro* migration was assessed by the scratch assay [79]. Attached cells grown to 95% confluence were serum starved for 6h, then treated with 10 ug/mL of mitomycin C (Sigma-Aldrich, cat#M4287) for 2h. The *in vitro* wound (scratch) was performed with a 200-μL pipette tip, medium was aspirated, cells were washed and incubated with complete media supplemented with mitomycin C. Digital images of two microscopic fields (40X magnification) were obtained from each well at 0 and 24h. The area devoid of cells between the migration fronts was determined using ImageJ 1.49q (NIH, USA). The average area in mm^2^ at 0h was subtracted from the average area determined at 24h to obtain the migrated area in mm^2^. Comparisons were always made between Control (CM PMBC-RPMI - indirect or PBMC incubated with RPMI - direct) and Test groups (CM PBMC-NOKsi/SCC-1/SCC-22B - indirect or PBMC primed with CM of epithelial cells - direct). Thus, the results indicate the effect of primed immune cells on the migration of non-malignant and malignant cells, normalized to the effect of non-primed PBMCs on the migration of non-malignant and malignant cells.

### Flow cytometry

PBMCs were cultured for 96h under different experimental conditions. Cells were counted, aliquoted and directly stained with 20μl (without fixation and permeabilization, as indicated by the manufacturer) with a CD8/CD69/CD3 antibody cocktail (BD FastImmune, BD Biosciences, cat# 340367) for analysis of activation. For immunophenotyping of the Th1/Th2/Th17 response, a different aliquot of cells was washed, fixed and permeabilized with Cytofix/Cytoperm (BD Biosciences) and stained with CD4-Percp-Cy5.5, 20μl test (cat# 341654), IFN-g-FITC, 5 μl/sample (cat# 554700), IL-4-PE-Cy7, 5 μl/sample (cat# 560672) and IL-17A- PE, 5 μl/sample (cat# 560486). Tregs were identified in a third aliquot of PBMCs that was washed, fixed and permeabilized with Human FOXP3 Buffer Set (BD Biosciences - cat# 560098), and stained with CD4-Percp-Cy5.5, 20 μl/sample (cat# 341654), FoxP3-AlexaFluor 488, 20 μl/sample (cat# 560047) and CD25-PE, 20 μl/sample (cat# 555432; all BD Biosciences). A minimum of 10,000 events were acquired in a FACSVerse flow cytometer and analyzed with FacSuite software (BD Biosciences).

### RT-qPCR

PBMCs were stimulated for 96h, centrifuged, washed and the RNA extracted using an affinity column system including DNAse treatment (RNAqueous-4PCR Total RNA Isolation kit, Ambion/ThermoFisher Scientific, cat#AM1914). cDNA was synthesized from 100 ng total RNA using random hexamers primers and reverse transcriptase (High Capacity cDNA Reverse Transcription kit, Applied Biosystems, ThermoFisher, cat#4368814). Real Time PCR was performed (TaqMan Fast Advanced Master Mix, Applied Biosystems, cat# 4444556) on a StepOne Plus thermocycler (Applied Biosystems). Data were analyzed by the relative quantification method ddCt with normalization to constitutive expression of GAPDH. Primers and TaqMan probes were pre-designed and optimized. Primers references: GAPDH - Hs99999905_m1, IFNG - Hs00989291_m1, IL4 - Hs00174122_m1, IL17A - Hs00174383_m1, TBX21 - Hs00203436_m1, GATA3 - Hs00231122_m1, RORC - Hs01076122_m1, ZBTB7B - Hs00757087_g1, IL10 - Hs0096162_m1, IL12A - Hs01073447_m1(Taqman Gene Expression Assays - Applied Biosystems).

### Data analysis

Four independent experiments were performed (two experiments with PBMCs from each donor) with at least three replicates in each experiment. All four independent results were grouped prior to statistical analysis with GraphPad Prism (GraphPad software),. A Student’s t-test was performed with a *P*-value of < 0.05 determined to be statistically significant.
